# Supplementary material for: HIVprotI: an integrated web based platform for prediction and design of HIV proteins inhibitors
Source: J Cheminform. 2018 Mar 9;10:12. doi: 10.1186/s13321-018-0266-y (PMC5845081; doi:10.1186/s13321-018-0266-y)
Supplement: Supplementary file 2 — Additional file 2. Source code of HIVProtI web server. [file 13321_2018_266_MOESM2_ESM.zip › HIVprotI_Source-code/hivprotI/weka-3-6-6/doc/weka/associations/package-frame.html]

weka.associations


weka.associations

Interfaces 
  
*Associator*
  
*CARuleMiner*

Classes 
  
AbstractAssociator
  
Apriori
  
AprioriItemSet
  
AssociatorEvaluation
  
CaRuleGeneration
  
CheckAssociator
  
FilteredAssociator
  
FPGrowth
  
FPGrowth.AssociationRule
  
FPGrowth.BinaryItem
  
GeneralizedSequentialPatterns
  
ItemSet
  
LabeledItemSet
  
Messages
  
PredictiveApriori
  
PriorEstimation
  
RuleGeneration
  
RuleItem
  
SingleAssociatorEnhancer
  
Tertius

Enums 
  
FPGrowth.AssociationRule.METRIC\_TYPE
